# Crocin protects against endoplasmic reticulum stress-related tubular injury in diabetic nephropathy via the activation of the PI3K/AKT/Nrf2 pathway

**DOI:** 10.22038/IJBMS.2023.73385.15942

**Published:** 2024

**Authors:** Guiying Wang, Jiuhon Deng, Zhou Hua

**Affiliations:** 1 Department of Nephrology, Shangyu People’s Hospital of Shaoxing, Shaoxing, China; 2 Department of Endocrinology, Second People’s Hospital of Pingyang County, Wenzhou, China; 3 Department of Nephrology, The People’s Hospital of Suichang County, Lishui, China

**Keywords:** Crocin, Diabetic nephropathy, Endoplasmic reticulum – stress, Kidney injury, PI3K/AKT

## Abstract

**Objective(s):**

Diabetic nephropathy (DN) is the main cause of end-stage renal disease, but the current treatment is not satisfactory. Crocin is a major bioactive compound of saffron with antioxidant and anti-endoplasmic reticulum stress (ERS) abilities used to treat diabetes. This study specifically investigated whether crocin has a regulatory role in renal injury in DN.

**Materials and Methods:**

The experiment was divided into control, (db/m mice), model (db/db mice), and experimental groups (db/db mice were intraperitoneally injected with 40 mg/kg crocin). Renal function-related indicators (Scr, BUN, FBG, UP, TG, TC, ALT, and AST) and oxidative stress-related indicators (ROS, MDA, GSH, SOD, and CAT) were assessed. The pathological changes of renal tissues were confirmed by HE, Masson, PAS, and TUNEL staining. The levels of ERS-related proteins (GRP78 and CHOP), apoptosis-related proteins, and PI3K/AKT and Nrf2 pathways-related proteins in renal tissue were detected.

**Results:**

In db/db mice, renal function-related indicators, apoptotic cells of renal tissues, the contents of ROS and MDA as well as the expressions of CHOP, GRP78, and Bax were increased, the degree of renal tissue damage was aggravated, while the contents of GSH, SOD, and CAT, as well as the protein levels of Nrf2, PARP, anti-apoptotic proteins (Mcl-1, Bcl-2, Bcl-xl) were decreased compared to the db/m mice. However, crocin treatment reversed the above-mentioned situation. The expressions of the PI3K/AKT and Nrf2 pathways-related proteins were also activated by crocin.

**Conclusion:**

Crocin inhibited oxidative stress and ERS-induced kidney injury in db/db mice by activating the PI3K/AKT and Nrf2 pathways.

## Introduction

Diabetic nephropathy (DN) is typically characterized by glomerular sclerosis, renal vascular degeneration, and tubulointerstitial fibrosis due to microangiopathy caused by chronic hyperglycemia (1). The pathological changes and mechanisms of DN are complex, and most of them show chronic progressive development, which can eventually progress to renal failure. At present, clinical treatment for DN mainly focuses on the control of hyperglycemia, blood pressure, blood lipids, inflammation, and anti-oxidants, but unfortunately, these treatments do not significantly curb the progression of DN (2). Therefore, finding new drugs to treat DN is of great significance for improving the therapeutic effect and prognosis of DN patients. 

Accumulated evidence shows that endoplasmic reticulum stress (ERS) exerts a crucial effect on the occurrence and development of DN (3). ERS refers to the dysfunction of the endoplasmic reticulum of stimulated cells, resulting in hindered protein processing, and accumulation of unfolded or misfolded protein. Moderate ERS is conducive to the stability of the intracellular environment, but persistent ERS will lead to impaired endoplasmic reticulum function and thus induce apoptosis (4). It has been found that hyperglycemia can start ERS, and ERS markers are up-regulated in the DN animal model and cell model, while inhibiting ERS is beneficial to improve DN (5, 6). The PI3K/AKT pathway exerts a crucial effect in regulating cell proliferation and survival. Study has shown that ERS inhibits the activation of the PI3K/AKT pathway to promote cell apoptosis, while activation of the PI3K/AKT pathway can reverse ERS-induced cell apoptosis (7). Hyperglycemia is a strong stimulus for oxidative stress and inflammation and also can down-regulate the activity of Nrf2 in diabetes (8). Studies also demonstrate that the function loss of Nrf2 can exacerbate ERS-induced cell apoptosis (9).

Crocin, the main bioactive component of saffron, has strong anti-oxidant and anti-inflammatory abilities and is used to treat diabetes, depression, heart disease, neurodegenerative diseases, and other diseases (10, 11). As an anti-oxidant fighter, crocin has been shown to inhibit ERS in myocardial cells damaged by ischemia-reperfusion, ovalbumin-induced mouse lung tissue, and high glucose-induced human umbilical vein endothelial cells (12-14). Furthermore, Abou-Hany *et al*. proposed that crocin can reduce the blood glucose level and oxidative stress index of streptozotocin-induced diabetic rats, but increase the insulin level and anti-oxidant defense capacity at the same time (15). However, the role of crocin as a protective agent against diabetic renal impairment and other diabetic complications is unknown.

Based on the above evidence, we speculate that crocin may improve DN by inhibiting ERS and related PI3K/AKT/Nrf2 pathways. Db/db mice and their control db/m mice are widely applied for drug screening and drug therapy research of DN (16, 17), which instigated us to study the beneficial effects of crocin on DN in db/db mice. 

## Materials and Methods

***Experimental Animals and ethics statement***

14-week-old C57BLKS/J db/m mice (n=6) and C57BLKS/J db/db mice (n=12) were supplied by Shanghai Slake Animal Laboratory Co. Ltd (Certificate No. SCXK (Hu) 2017-0005). The room temperature was maintained at 21 ± 0.5 ℃ and humidity was maintained at 45–50% with a normal period (12 hr light/12 hr dark). Our research was approved by the Ethics Committee of Hangzhou Eyong Biotechnological Co., Ltd. Animal Experiment Center (Certificate No. SYXK (Zhe) 2020-0024), and in accordance with the guidelines of the China Council on Animal Care and Use. 

***Treatment***

The experiment was divided into three groups: control group (db/m mice), model group (db/db group), and experimental group (db/db + crocin group). Two weeks later, C57BLKS/J db/db mice were randomly grouped as follows: db/db group and db/db+crocin group (6 per group). Mice in the db/db group received intraperitoneal injections of normal saline, while mice in the db/db+crocin group received intraperitoneal injections of crocin (YZ-0329, Solarbio, China) dissolved in normal saline (40 mg/kg/d) **(**18**)**. After 8 weeks of continuous drug treatment of the mice, all mice were euthanized by overdose of sodium pentobarbital. Kidney and blood samples were collected for further analysis. 

***Evaluation of renal function***

The fasting blood glucose (FBG) of each group was determined by orbital blood sampling using a blood glucose meter (580, Yuwell, China). Twenty-four-hour urine sample collection was performed using a metabolism cage, followed by urinary protein (UP) extraction by Liquid Protein Extraction Reagent (P1255, Applygen, China) and quantitation by the BCA kit (P1513, Applygen, China). The collected blood samples were centrifuged at 3000 r/min for 10 min to obtain serum. The levels of serum creatinine (Scr), blood urea nitrogen (BUN), fasting blood glucose (FBG), urinary protein (UP), triglyceride (TG), total cholesterol (TC), alanine aminotransferase (ALT), and aspartate aminotransferase (AST) were detected using an automatic biochemical analyzer (AU680, Beckman, USA). 

***Kidney histopathology***

The collected kidney tissue was subjected to fixing with 10% neutral formalin solution (E672001, Sangon, China). Following embedding in paraffin, the tissues were sliced into 5-μm-thick sections. . After being routinely dewaxed and hydrated, the sections were stained with a hematoxylin and eosin (HE) kit (G1003, servicebio, China), Masson kit (G1006, servicebio, China), and Periodic Acid-Schiff (PAS) stain kit (G1008, servicebio, China). The sections were dehydrated and transparentized followed by sealing with neutral balsam mounting medium (G1405, servicebio, China). The pathological changes and fibrosis degree of mouse kidney tissue were observed under a microscope (BX53M, Olympus, Japan, 400×).

***Terminal deoxynucleotidyl transferase-mediated deoxyuridine triphosphate nick end labeling (TUNEL) staining***

TUNEL staining of kidney tissue was conducted using the TUNEL Apoptosis Detection Kit (G1507, Servicebio, China). After being routinely dewaxed and hydrated, the sections were subjected to proteinase K repairing followed by cultivating with 3% hydrogen peroxide (H_2_O_2_). After washing with PBS, the section was incubated with TdT incubation buffer and then, reacted with Streptavidin-HRP working solution. The sections were developed by DAB solution, and a hematoxylin staining solution was applied for nuclear staining. Afterwards, the sections were dehydrated and transparentized followed by sealing. The apoptosis cells were observed under a microscope. 

***Immunohistochemistry and immunofluorescence***

After being routinely dewaxed and hydrated, the sections were subjected to antigen repair using citric acid (PH6.0) antigen repair solution (G1202, Servicebio, China). After treatment with 3% H_2_O_2_, the sections were subjected to blocking with BSA (abs9157, absin, China). For immunohistochemistry, the sections were probed with primary antibodies: anti-GRP78 (AF5366, Affinity, USA) and anti-CHOP (DF6025, Affinity, USA) followed by reacting with HRP-labeled secondary antibodies (S0001, Affinity, USA). The sections were developed by DAB solution and a hematoxylin staining solution was applied for nuclear staining. For immunofluorescence, the sections were incubated with primary antibodies: anti-GRP78 (AF5366, Affinity, USA) and anti-CHOP (DF6025, Affinity, USA) followed by reacting with Alexa Fluor® 488-labeled secondary antibodies (ab150077, Abcam, USA). DAPI solution (G1012, servicebio, China) was applied for nuclear staining. Finally, the results were observed by a microscope (200 or 400×) or a fluorescence microscope (400×, Nikon ECLIPSE, Nikon, Japan). 

***Measurement of oxidative stress-related markers***

The levels of oxidative stress-related marker reactive oxygen species (ROS), malondialdehyde (MDA), glutathione (GSH), superoxide dismutase (SOD), and catalase (CAT) were measured using corresponding kit (MM-043700M1, MM-0388M1, MM-0758M1, MM-0389M1, and MM-44125M1) based on the instructions of the manufacturer (Meimian, China). Briefly, the kidney tissue (0.5 g) was ground in liquid nitrogen and potassium phosphate buffer. The homogenate was centrifuged to collect the supernatant. The supernatant was added to the plate pre-coated with antibody followed by reacting with enzyme-labeled antibody, chromogenic solution, and stop solution. The wavelength at 450 nm was measured using a microplate reader.

***Western blot***

Tissue lysis was conducted using RIPA buffer (PC104, epizyme, China) to extract the total protein after the quantification was determined using a BCA kit (ZJ101, epizyme, China). The protein extract was subjected to SDS-PAGE electrophoresis and then, transferred to PVDF membrane (FFP24, Beyotime, China). After blocking, the membranes were probed with primary antibodies and then reacted with secondary antibodies. GAPDH was used as an internal reference. The antibodies are shown in Table 1. The intensity of band signals was visualized using an ECL luminescence reagent (P1000, Applygen, China) equipped with a ChemiDocTM XRS plus imaging System (Bio-Rad, USA).

***Statistical analysis***

The measurement data were presented as mean ± standard deviation, and one-way analysis of variance (ANOVA) was adopted for the comparison among the multiple groups. For pairwise comparison between groups, the Tukey test was used for homogeneity of variance, and the Kruskal-Wallis H test was used for heterogeneity of variance. All statistical analyses were implemented with GraphPad 8.0 software, and *P*-values less than 0.05 were considered statistically significant.

## Results

***Crocin alleviated renal dysfunction of db/db mice***

**Figure 1:**
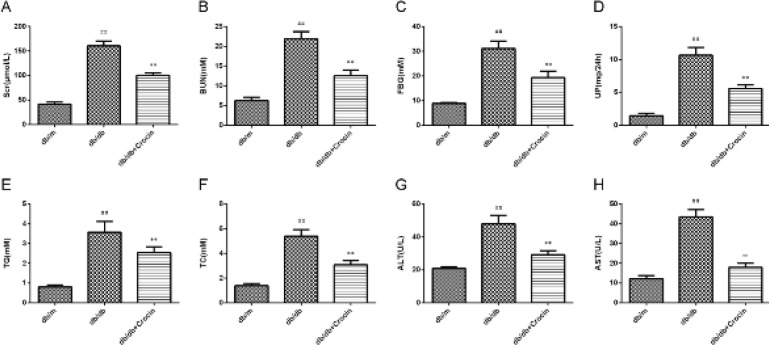
Content of serum creatinine (Scr) (A), blood urea nitrogen (BUN) (B), fasting blood glucose (FBG) (C), urinary protein (UP) (D), triglyceride (TG) (E), total cholesterol (TC) (F), alanine aminotransferase (ALT) (G), and aspartate aminotransferase (AST) (H) was measured in db/m mice and db/db mice treated with or without crocin

Biochemical indicators for the three groups of mice are depicted in Figure 1A-H. Compared with db/m mice, the levels of Scr, Bun, FBG, UP, TG, TC, ALT, and AST in db/db mice were significantly increased (*P*<0.01), while abnormalities of these biochemical indicators tended to be normal after administration of crocin (*P*<0.01). 

***Crocin reduced kidney tissue damage and apoptosis of db/db mice***

**Figure 2:**
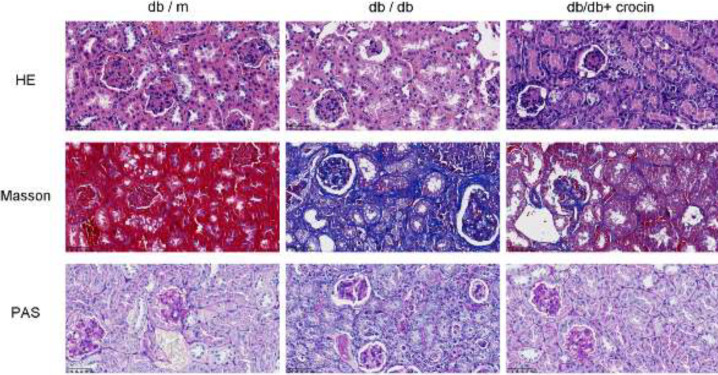
Histopathological changes in mouse kidney (A) H&E staining. (B) Masson staining. (C) PAS staining

**Figure 3:**
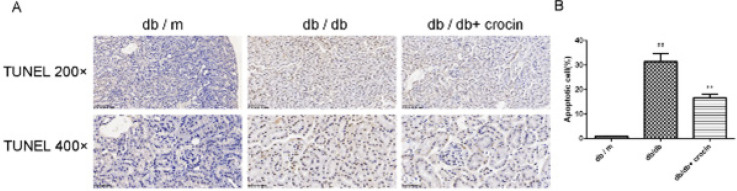
Apoptosis cells of mouse kidney tissue

Histopathological staining was performed to identify renal tissue damage (Figure 2). Compared with db/m mice, db/db mice had iglomerular hypertrophy, widespread proliferation of glomerular mesangial cells, and swelling of glomerular and renal tubular epithelium. However, after administration of crocin, the glomerular volume was significantly reduced, with a small number of mesangial cells proliferated, and renal fibrosis was also improved. In addition, crocin administration also reduced the apoptotic cells in the kidney tissue of db/db mice (Figure 3A-B, *P*<0.01). 

***Crocin decreased the levels of ERS-related proteins in the kidney of db/db mice***

**Figure 4:**
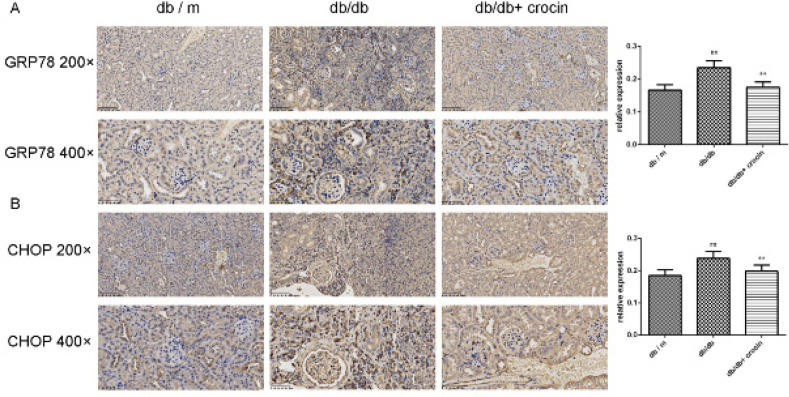
Immunohistochemical analysis of the expression of GRP78 (A) and CHOP (B) in mice kidney tissue

**Figure 5:**
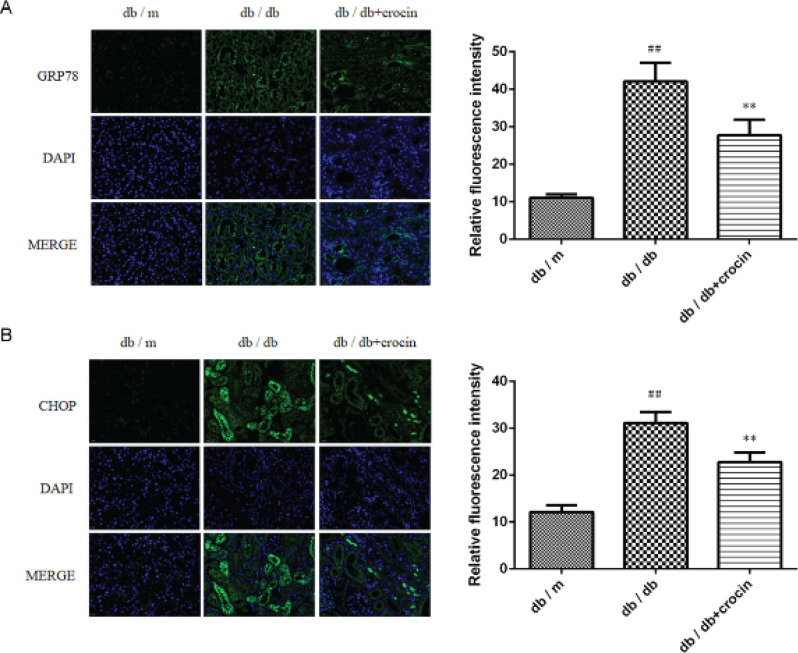
Immunofluorescence analysis of the expression of GRP78 (A) and CHOP (B) in mouse kidney tissue

The levels of ERS-related proteins GRP78 and CHOP were evaluated. As depicted in Figure 4A-B, the protein levels of GRP78 and CHOP in the kidney tissue of db/db mice were higher than those of db/m mice, while the treatment with crocin brought the elevated GRP78 and CHOP back to normal levels (*P*<0.01). The results of immunofluorescence staining were consistent with those of immunohistochemistry, that is, crocin suppressed the expression of GRP78 and CHOP in kidney tissue of db/db mice (Figure 5A-B, *P*<0.01). 

***Crocin attenuated oxidative stress of db/db mice***

**Figure 6:**
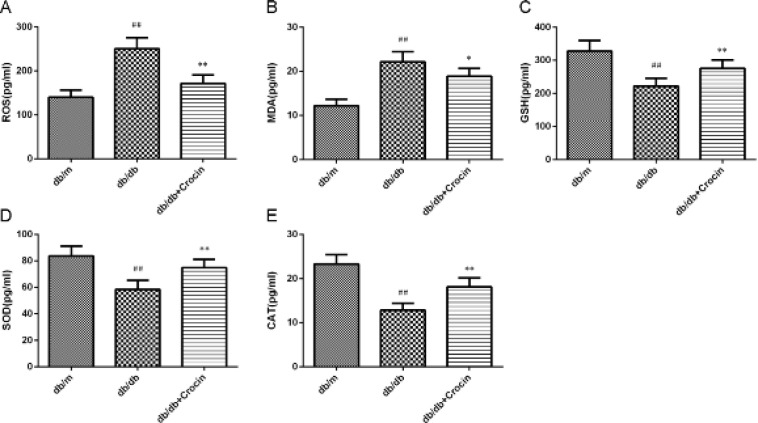
Content of reactive oxygen species (ROS) (A), malondialdehyde (MDA) (B), glutathione (GSH) (C), superoxide dismutase (SOD) (D), and catalase (CAT) (E) in mouse kidney tissue homogenate was measured

The content of oxidative stress-related indicators in mouse kidney homogenate was detected by the kit. The increased contents of ROS and MDA, as well as the decreased contents of GSH, SOD, and CAT, were observed in the db/db group, while these abnormal oxidative stress-related indicators tended to be normal after administration of crocin (Figure 6A-E, *P*<0.01).

***Crocin activates Nrf2 and PI3K/AKT signaling pathways and regulates the expression of apoptosis-related proteins***

**Figure 7:**
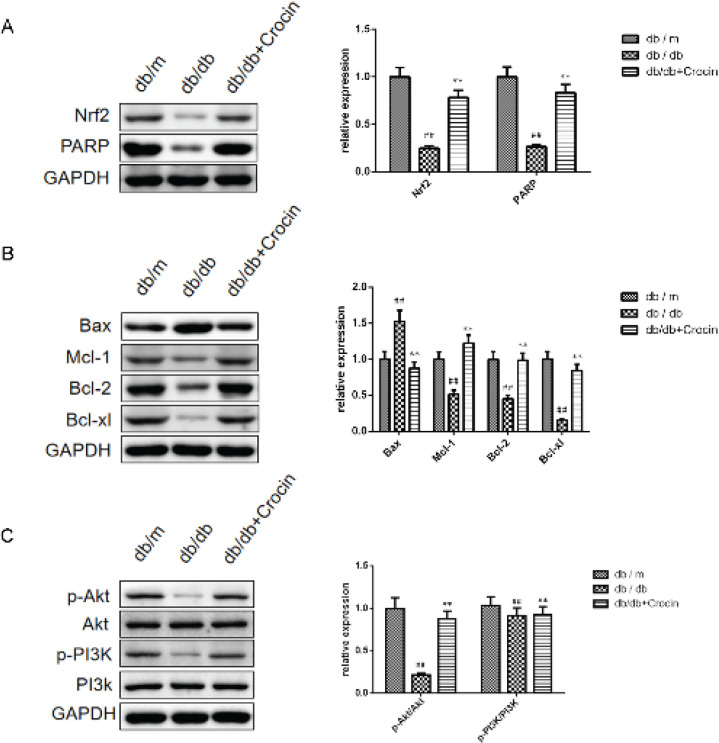
Protein levels of mice kidney tissue were measured by western blot. (A) Protein levels of Nrf2 and PARP. (B) Protein levels of Bax, Mcl-1, Bcl-2, and Bcl-xl. (C) Protein levels of p-Akt/Akt and p-PI3K/PI3K

As shown in Figure 7A-C, the db/db group showed down-regulated expression of Nrf2, PARP, Mcl-1, Bcl-2, Bcl-xl, p-AKT/AKT, and p-PI3K/PI3K, and up-regulated expression of Bax compared to these in db/m mice. After treatment, these expressions were reversed by crocin (*P*<0.01). 

**Table 1:** Antibodies used in Western blot

Name	Catalog	Molecular weight	Manufacturer
Nrf2	ab92946	70 kDa	abcam, UK
PARP	ab191217	113 kDa	abcam, UK
Bax	ab32503	21 kDa	abcam, UK
Mcl-1	ab32087	37 kDa	abcam, UK
Bcl-2	ab182858	26 kDa	abcam, UK
Bcl-xl	ab32370	26 kDa	abcam, UK
p-Akt	AF0016	56 kDa	Affinity, USA
Akt	Ab8805	56 kDa	abcam, UK
p-PI3K	AF3242	54 kDa	Affinity, UK
PI3K	ab86714	85 kDa	abcam, UK
GAPDH	ab8245	36 kDa	abcam, UK
goat anti rabbit IgG H&L (HRP)	ab205718	—	Abcam, UK

p-Akt: phosphor-Akt. GAPDH: Glyceraldehyde-3-phosphate dehydrogenase

data are expressed as mean±SD, n=6). ##*P*<0.01 vs db/m group. **P<0.01 vs db/db group

Data are expressed as mean±SD, n=3, 400×)

TUNEL staining. Data are expressed as mean±SD, n=3, 200 or 400×. ##*P*<0.01 vs db/m group. **P<0.01 vs db/db group

Data are expressed as mean±SD, n=6, 200 or 400×). ##*P*<0.01 vs db/m group. **P<0.01 vs db/db group

Data are expressed as mean±SD, n=6, 400×. ##*P*<0.01 vs db/m group. **P<0.01 vs db/db group

Data are expressed as mean±SD, n=6. ##*P*<0.01 vs db/m group. *P<0.05, **P<0.01 vs db/db group

Data are expressed as mean±SD, n=6. ##*P*<0.01 vs db/m group. *P<0.05, **P<0.01 vs db/db group

## Discussion

DN has been the main cause of end-stage renal disease, but the current treatment is not satisfactory. Recent studies have shown that ERS plays a key role in the occurrence and development of DN (19). Therefore, alleviating ERS in DN has become a hot research topic in the treatment of DN. Although crocin has the effect of inhibiting ERS, whether it can treat DN by inhibiting ERS is not completely clear. In this study, the function of crocin in DN was clarified through animal experiments, providing a new direction for the treatment of DN.

We first determined crocin’s effect on renal function in db/db mice by measuring the renal function-related indicators (Scr, BUN, FBG, UP, TG, TC, ALT, and AST). Scr and BUN are specific indicators for clinical judgment of renal function. When the kidney is damaged or the glomerular filtration capacity is decreased, the exclusion of BUN and Scr is decreased, and the content of BUN and Scr in the blood is increased. The most basic mechanism of DN is renal hemodynamic changes caused by abnormal glucose metabolism. Therefore, stabilizing blood glucose (FBG) is the key to the prevention and treatment of DN (20). With the progression of DN, albumin can be excreted in the urine of patients with DN, so the content of UP is closely related to the progression of DN (21). In addition, patients with DN often have lipid metabolism disorders (TG and TC are lipid metabolism indicators), excessive lipids can be deposited on the glomerular basement membrane, stimulating the proliferation of mesangial cells damage the kidney (22). AST and ALT are transaminases in the liver and kidney, which are released intracellularly when tissues are damaged (23). In our study, the elevated renal function-related indexes (Scr, Bun, FBG, UP, TG, TC, ALT, and AST) in db/db mice were all reduced after crocin administration, indicating that crocin can improve the renal function of db/db mice, which is also consistent with the previous report that crocin can improve the indexes of liver and kidney damage (24, 25).

Renal pathomorphological detection can directly reflect the degree of pathological damage of the disease and is an important indicator for evaluating the treatment effect. Pathological manifestations of DN are glomerular hypertrophy, widened mesangial area, basement membrane thickening, matrix hyperplasia, tubular atrophy, and renal interstitial fibrosis with inflammatory cell infiltration (26)­. Moreover, more apoptotic cells will appear in the kidneys of DN mice (27). Previous studies have proposed the renal protective function of crocin, including reducing ischemia-reperfusion-mediated apoptosis of kidney tissue, improving high glucose-induced renal fibrosis, and protecting gentamicin-induced glomerular atrophy (28-30). Consistent with previous studies, we found that the kidney injury of db/db mice was improved after crocin administration. 

GRP78 is a chaperone on the ER membrane. When unfolded or misfolded proteins aggregate in the ER, GRP78 is dissociated from the transmembrane receptor protein to initiate the ERS signal transduction pathway. ERS mainly induces apoptosis by increasing the transcription level of CHOP. A large number of animal experiments proved the up-regulation of GRP78 and CHOP in renal tissue of the DN animal model (31, 32). Of note, crocin can inhibit the expression of GRP78 and CHOP in both Alzheimer’s disease rat model and myocardial ischemia-reperfusion model mice (12, 33). To determine the regulatory effect of crocin on ERS in DN, we detected the expression of GRP78 and CHOP, two signature proteins of ERS in db/db mice. Similarly, we found that crocin inhibits the expression of GRP78 and CHOP in the kidney of db/db mice, suggesting that crocin plays a renal protective role by inhibiting ERS.

ERS can interact with oxidative stress to mediate DN hemodynamic disorders and renal metabolic disorders (34). In addition, the potent anti-oxidant properties of crocin have been well documented. To this end, we tested the biochemical indicators of oxidative stress, ROS, anti-oxidant enzymes (GSH, SOD, and CAT), and the end product of lipid peroxidation (MDA). In our study, the down-regulated expression of anti-oxidant enzymes and the increased ROS and MDA contents in db/db mice were both reversed by crocin, which is also consistent with the previously reported effect of crocin on reducing lipid peroxidation and increasing the expression of anti-oxidant enzymes under anti-oxidant stress (35). Of note, ROS is also the upstream signal molecule that triggers the ERS-mediated apoptosis pathway (36). Therefore, our results may indicate that crocin played a renal protective role by resisting oxidative stress to inhibit ERS. 

In addition, we characterized the effect of crocin on DN at the molecular level. Nrf2’s primary function is to coordinate the intracellular oxidative stress response, and its activation has been shown to protect DN, while PARP recognizes DNA damage to promote DNA repair (37-39). Anti-apoptotic proteins (Mcl-1, Bcl-2, and Bcl-xl) can bind to pro-apoptotic protein (Bax) to promote cell survival (40). It was reported that ROS in DN can inactivate PI3K/AKT and promote the pathogenesis of DN (41). Importantly, crocin has been shown to activate the Nrf2 pathway and PI3K/AKT pathway to inhibit apoptosis and ERS for neuroprotective and cardioprotective purposes (12, 42). Consistently, we found that crocin also plays a renal protection role by activating the Nrf2 and PI3K/AKT pathways to inhibit apoptosis. It has been proved that ERS can mediate apoptosis of podocytes through CHOP (43). Therefore, we speculate that crocin may inhibit ERS-mediated apoptosis by inhibiting the Nrf2 pathway and PI3K/AKT pathway, thereby improving kidney injury.

## Conclusion

In summary, this study showed that crocin inhibited oxidative stress and ERS-induced kidney injury in db/db mice, the activation of the PI3K/AKT and Nrf2 pathways was related to the underlying mechanism of crocin. This study hopes to provide a clue for developing a novel strategy for the treatment of DN.

## Authors’ Contributions

G W and Z H designed the experiments; J D performed experiments and collected data; G W discussed the results and strategy; Z H supervised, directed, and managed the study; G W drafted the manuscript; Z H critically revised and edited the article; G W, J D, and Z H approved the final version to be published.

## Funding

Not applicable.

## Conflicts of Interest

The authors declare that they have no competing interests.
